# Clinical Pathology and General Practice

**Published:** 1957-10

**Authors:** H. J. Gibson

**Affiliations:** Area Pathologist, Bath Clinical Area


### CLINICAL PATHOLOGY AND GENERAL PRACTICE

BY

H. J. GIBSON, M.D., D.P.H.
*Area Pathologist, Bath Clinical Area*

One of the most significant medical trends in England today is the movement towards treatment at home of many patients formerly regarded as hospital cases. Treatment by antibiotic, by vitamin or by anticoagulant can be carried out at home by the keen family doctor as efficiently as in hospital in those cases which do not require skilled nursing or 24-hour supervision. The saving of expense to the State is obvious and for this reason the change is part of current policy of the Ministry of Health. How far the tendency will go depends on the progress of research into the cause and cure of degenerative cardiovascular diseases, neoplasms and rheumatism. In future the hospital wards may become increasingly places for investigation and for initiation and stabilization of a treatment régime which can be continued by the family doctor at home. The process of transition with disappearance of hospital beds is seen at present in varying degrees for infectious diseases, venereal diseases and tuberculosis. It need hardly be said that this trend towards domiciliary treatment is welcomed by all who have the welfare of general practice at heart and most by the general practitioner himself. With full access to special departments his status at once becomes that of a scientific physician able to practise the medicine he learned in his teaching hospital.

An essential pre-requisite for the management of cases at home is that full laboratory facilities should be available. Initial diagnosis may then be made with certainty, treatment may be guided by laboratory findings, and the progress of the case followed in an objective way by the use of appropriate laboratory tests. Accurate prognosis and assessment of cure can be placed on a safe footing.

### HOW TO USE THE SERVICE

This paper explains how a practitioner can make best use of the services of the laboratory in the day-to-day running of his practice. The service is now fully available in all provincial and most teaching centres, and all that is required, is that the doctor should be able to take specimens and deliver them without delay to the nearest laboratory. Even this small effort is unnecessary with ambulant patients who can attend the laboratory. For them an appointment must be made as certain times are set aside at which practitioners' cases can be seen. Patients arriving at other times may have to wait, or, what is more important, there may be delay in examining specimens with consequent inaccuracy of results. Where the case is not an urgent one, many practitioners find it convenient to complete the laboratory "Request Form" stating on it "Please make appointment" and send it to the laboratory secretary who can then communicate directly with the patient and make an appointment for his attendance. Thus having posted the request form the practitioner need only await the arrival of the report.

Laboratories differ in the arrangements made for general practitioners' work and the first step is to ascertain the name of the nearest or most conveniently situated laboratory and to make personal contact with the pathologist and his staff. Details may then be discussed of times and methods. Outfits for specimen collection may be obtained and hints given which will smooth out any difficulties which may arise. Pathologists often have a wide field of subsidiary laboratories and other hospitals to visit and it is well to make the acquaintance of chief and senior technicians in the various departments. These men are highly expert in all branches of their work and are able to give practical advice on both technical and theoretical points. They are available at any time during laboratory hours when the medical staff may be inaccessible.

It is very desirable that each practitioner should deal always with the same laboratory even if more than one is conveniently situated. Records are kept of each patient and reference to them by the pathologist making the report may be most helpful in elucidating a problem of diagnosis. To have such records scattered over a number of laboratories is as detrimental to the patient's interests as would be his having two or more family doctors.

The pathological services are already widely used by general practitioners. In Bath, where these services have been freely offered since the appointed day, the proportion of G.P. work is steadily rising and now accounts for 12 per cent of the total. If all general practitioners used it as enthusiastically as the few who co-operate most fully with the laboratory the proportion would be far higher. Work from practitioners is welcomed by all pathologists who find it most interesting. After nearly nine years of the National Health Service the author has never encountered any abuse of this service. The investigations requested are relevant, usually simple and the "specific problem" is usually one in which the laboratory can help. In many cases clues obtained from a simple test can be followed up on the material sent and a definite diagnosis made without further reference to the practitioner. We have rarely found general practitioners employing the regrettable "drag-net" technique in which a mass of unrelated investigations are ordered in the hope that some clue to diagnosis may emerge. As with "drag-nets" elsewhere the most likely finding is an "old boot" in the form of a useless and misleading minor abnormality. Pathological requests should always arise out of the clinical study of the patient and one line of investigation should be exhausted before another is commenced.

### SOME PITFALLS

To those who may be unfamiliar with the interpretation of laboratory findings it cannot be too strongly emphasized that *no* diagnosis should be founded on a single pathological result if it is at variance with the overall clinical picture. Examples of this pitfall are legion. Thus a very high "blood urea" may be found in a blood containing Wintrobe's ammonium oxalate mixture as anticoagulant if the common Nesslerization method is used. Alarming blood-sugar values have been found in blood taken with a syringe recently used for injecting glucose solution. Consternation was caused many years ago when a patient in a rheumatic-diseases hospital, who was a pillar of one of the sterner religious sects, was found to have a positive Wassermann reaction. Reviewing his case in the light of recent knowledge, it is almost certain that he had disseminated lupus erythematosus in which a biological false positive Wassermann reaction is a recognized finding, as it is of course in some cases of glandular fever and atypical virus pneumonitis. In all cases of this kind when doubt exists, the test should be repeated and the findings discussed with the pathologist.

Another common source of error arises from not approaching the investigation of a problem case in a systematic way. It is better to begin with a Blood Sedimentation Rate, blood count and urine. The more spectacular tests can come later. It is astonishing how often a rare and puzzling syndrome resolves itself into an attack of simple pyelitis when the urine is examined! Clotted blood was sent for Brucella agglutination test from a farmer with unexplained fever; it was positive, and much valuable time was lost before the case was reinvestigated and found to be one of generalized tuberculosis. The Brucella antibodies were presumably the result of infection previously acquired during his work in handling infected cattle.

The clinician should not expect all laboratory results to agree fully with the classical patterns associated with specific diseases. Many factors modify the findings including especially previous treatment. For this reason a note of treatment given should always be made on the request form if it is likely to modify the laboratory findings in any way. It is common now to find a purulent cerebrospinal fluid to be sterile on culture and negative blood cultures do not exclude an active infective endocarditis. Carcinoma of the stomach usually increases the B.S.R. but a normal result may be found in scirrhous non-ulcerating cases and also in some cases where the liver is involved. In presence of jaundice or other evidence of liver damage the B.S.R. is suspect. A rapid rate is valid but a normal rate excludes nothing. Many similar examples could be given.

As the old killing diseases are being mastered, others, once rarities, are taking their places. It is necessary, however, to preserve a sense of proportion. Polyarteritis nodosa and disseminated lupus erythematosus are undoubtedly more common than before, but they are not the solution of all diagnostic problems. It is still necessary to base investigation on the assumption that common diseases are more likely to be present and should be excluded first. The residuum of resistant and undiagnosed conditions will include the rarities which tax to the uttermost the resources of haematology, bacteriology, biochemistry, and histology to establish the diagnosis. Such cases should be in hospital.

### COLLECTION OF SPECIMENS

*Blood collection*

Venous blood is necessary for most biochemical tests and is preferable for haematological examination. With practice arm puncture is the most rapid method and the least irksome to the patient. A piece of rubber tubing 18 inches long makes a good tourniquet. A wide-bore needle (No. 19 B.W.G.) is preferable (with short bevel). Needles must be completely sterile and may be obtained autoclaved in glass tubes from the laboratory. In view of the risk of transmitting hepatitis, syringes must be well boiled after use.

An alternative method is to allow the blood to flow, under venous pressure, from the needle through a short length of translucent polythene tubing into the test-tube or bottle in which the specimen is being collected. A double-bored rubber stopper exactly fitting the container has two short lengths of glass tube through it. To one the polythene tube is connected, the other being an air-vent. An autoclaved needle is attached just before use. This method has the following advantages: there is virtually no risk of hepatitis, and it is a "one hand" method. The patient's arm is grasped firmly and the needle inserted until the blood is seen to flow through the polythene tube into the container. The fact that one's grasp of the arm need never be relaxed makes it an ideal method of taking blood from children. Finally it is practically unbreakable and can be easily cleaned under a tap. Complete sterility—except for the needle—is unnecessary.

Screw-cap bottles of the U.G.B. 2-dram vial type or the larger "Universal Container" are now widely used owing to their strength. A plentiful supply, some containing dry anticoagulant and some plain, should be kept at hand. The anticoagulant which we favour is E.D.T.A. (ethylene diamine tetra acetate) or Sequestrene. This is excellent for preserving the blood cells for counts and for reliable B.S.R. determination while the plasma gives excellent results in agglutination tests. It is not suitable for Wassermann Reaction or Khan tests. Most biochemical tests except serum calcium and prothrombin content estimation can be done on E.D.T.A. blood. Serum-calcium estimation requires clotted blood and for prothrombin determination 0·5 ml. of 3·8 per cent. sodium citrate is present in the tube, to which 4·5 ml. of blood are added and shaken. If both clotted and unclotted blood are required and the needle and tube technique is used, blood is taken directly into the plain bottle and part transferred to the anticoagulant bottle which is at once well shaken.

Little need be said about the other specimens such as faeces, urine, exudates and swabs, except to mention the newer transport media, such as Stuart's, in which exudates on swabs may be preserved for considerable periods. It is especially valuable for vaginal swabs because the gonococcus and trichomonas vaginalis remain viable.

For details of lumbar puncture and methods of tapping the serous cavities a larger work such as *Clinical Pathology in General Practice* published by the *British Medical Journal* should be consulted.

### COMPLETION OF REQUEST FORMS

There is a tendency to regard these as unnecessary paper-work. No fallacy could be more dangerous. A busy laboratory receives some hundreds of specimens in a day and unless they are carefully labelled (name, initials and date) and accompanied by a fully completed request form, serious and even tragic mistakes may be made. Unlabelled specimens cannot be accepted in blood-transfusion work and in other cases we mark the report "Specimen unlabelled" and no responsibility can be taken for errors of identification. On the request form full Christian names should be given and date of birth is a better identification feature than age alone. The date and time of taking the specimen will show the pathologist what tests are possible and what allowance must be made for deterioration. Space is always provided for clinical details. A long essay on the case is not called for. A few well-chosen words will indicate the duration of illness, the salient clinical features, any tentative diagnosis clinically suggested and any specific treatment given. Thus "3/12 loss of weight, dyspepsia, afebrile? Ca. stomach? Anaemia. Imferon given" provides the pathologist with a background against which he can interpret his findings and possibly suggest or carry out further tests. Without these few words he is working in the dark. The assistance given to the practitioner by a pathological report is often in proportion to the information which he gives the laboratory. Time spent on the request form is well spent.

### NOTES ON MORE COMMON METHODS AND APPLICATIONS OF CLINICAL PATHOLOGY

### *Anaemia*

Where the symptoms point to anaemia, leukaemia or other blood disease a vial of at least 3 ml. of well-shaken unclotted blood should be sent together with a dried blood film. The drop of blood in the needle may be used for making this film. For postal specimens the importance of a well-made film cannot be over-emphasized. Every practitioner should secure a supply of microscopic slides and practise until he can make a really good film. This must be quite dry before it is wrapped in paper and enclosed with the bottle. The name may be written in ink over the thick end of the blood film. A well-made film alone can often be far more informative than a bottle of ageing blood.

If the blood shows an iron-deficiency anaemia of low colour index the question of its cause arises. A B.S.R. estimation can be very helpful, a definite increase indicating that there is some underlying disease process such as infection or neoplasm to which anaemia is secondary. A leucocytosis or leucopenia may point to infection, or abnormal white cells to leukaemia. In every case of severe unexplained iron-deficiency anaemia, a faeces specimen should be sent for occult blood test before and after a three-day period on a white diet. Blood loss is by far the commonest cause of this type of anaemia and treatment must be directed to the bleeding point rather than the blood. Follow-up examinations only call for haemoglobin estimation at intervals of 2–4 weeks. Full blood counts are unnecessary when a diagnosis has been established.

If the anaemia is of macrocytic type with high colour index a complete diagnosis cannot be made on peripheral blood examination alone. A test meal is required to determine whether there is achlorhydria and sternal marrow examination is also necessary. For the general practitioner a Diagnex (Squibb) tubeless test meal is able to show whether free hydrochloric acid is present or absent. It involves the oral administration of the contents of two small cachets and the collection of three timed urine samples. Estimation of quinine in the urine indicates whether free acid is present or absent. Full instructions are given with each outfit.

A complete diagnosis requires in addition sternal puncture and a faecal fat balance test. If it is not possible for the patient to go into hospital and in presence of *typical* clinical and peripheral blood pictures of pernicious anaemia including achlorhydria and normal B.S.R. then many practitioners are satisfied to give a trial dose of Vitamin B12 and have the blood examined for a reticulocyte count in 5 to 10 days. If this response is satisfactory, treatment may be continued with confidence. If the anaemia does not respond, full investigation in hospital is necessary, but by then the vitamin given may have modified the marrow picture. So it is clearly best to have the full investigation made before any treatment is given. On no account should the therapeutic test with folic acid be made because in true Addison's anaemia (true P.A.) folic acid precipitates devastating nervous involvement. It is possible but not desirable to carry out sternal puncture as an out-patient procedure. It alarms the nervous patient and preliminary sedation is desirable.

If the clinical, radiological or white-cell pictures point to leukaemia or neoplasm in bone, sternal marrow examination is a *sine qua non*. The practitioner should remember that leukaemia is becoming far more common than in the past and that its clinical presentation is often highly atypical.

*Ante-Natal Blood Testing*

Blood from every expectant mother should be examined early in pregnancy for three reasons, first to determine whether anaemia is present, secondly to carry out Wassermann and Kahn tests, thirdly to ascertain the patient's Rhesus (Rh.) blood group. The A B O group is also determined but this is of less importance, for it does not obviate the need for confirmation of the patient's group and a full cross match if the mother requires blood transfusion at a later date. A special form must be completed to accompany each ante-natal specimen because the extent and interpretation of the pathologists' investigation depends on the history of previous pregnancies and blood transfusions. If the patient cannot attend at the laboratory, clotted and Wintrobe-oxalate blood samples (5 ml. of each) are required. Haemolytic disease of the new born complicates 1 in 200 pregnancies and it is essential that the possibility of its existence be determined before labour, and the mother transferred to a hospital where facilities for immediate and repeated exchange transfusion of the baby are available should this prove necessary. Demonstrations of antibodies in maternal serum is the only way of selecting such cases.

If the mother is found to be Rh. negative at the first test, then, if she be a primiparous woman without history of previous blood transfusion, no further test is necessary. A second sample of clotted blood must be sent at the thirty-fourth week from all Rh. negative primiparous women with a history of having received a blood transfusion and from all multiparous Rh. negative mothers. This sample is tested for Rh. antibodies. If they are found the husband's blood should be sent for examination.

If the mother, whether primiparous or multiparous, is found to be Rh. positive, in the absence of a history of blood transfusion or of clinical evidence of haemolytic disease in a previous baby, no further test is necessary. If such a history is present the mother's blood should be retested at the thirty-fourth week for unusual immune antibodies. If they are found the husband's blood should be examined. It should be noted that the husband's blood is only examined when the mother's serum contains antibodies or where there has been a history of unexplained still-birth or a jaundiced baby. In other cases it causes alarm and is not really helpful.

For the diagnosis of haemolytic disease in the new born, 2 to 5 ml. each of oxalated and clotted cord blood should be sent at once to the laboratory where the blood count and haemoglobin may be determined and the red cells examined for evidence of sensitization by antibody.

*Blood Sedimentation Test.*

Among the simple laboratory procedures, valuable in many difficult cases, the B.S.R.

estimation occupies a unique place. The only cause of physiological increase is pregnancy and a raised rate is otherwise an indication of organic disease. It is not specific for any one disease or group of diseases. It is a reflection of some change in plasma protein constitution, though this change varies from case to case. In acute diseases fibrinogen is often increased while in chronic disease $\alpha$ and $\gamma$ globulin may be raised. It is sometimes stated that a raised rate indicates infection, but this is not so for Gout, leukaemia and coronary thrombosis are non-infective conditions in which the sedimentation rate is often high. On the other hand in superficial catarrhal infections and in unlocalized systemic infections by bacteria and viruses the rate may be normal or nearly so. The rate may be high in liver disease when there is usually some change in plasma protein. Unclotted blood (oxalate or E.D.T.A.) as used for the blood count is quite suitable for the B.S.R. provided that it arrives at the laboratory within three hours of taking. In view of the value of the test a number of country practitioners remote from the laboratory have found it worth while to acquire a simple rack and a number of Westergren tubes. These are obtainable commercially at all laboratory supply houses.

The procedure is simple. Have at hand a bottle of 3·8 per cent. sodium citrate solution. Fill the pipette to the 150 mm. mark (i.e. one quarter full) with citrate solution and place it in a small test-tube. To this add one pipette full to the O mark with blood from the specimen before it is sent to the laboratory for the count. Blood and citrate are mixed and the pipette is then filled with diluted blood to the mark and placed vertically in the stand. A simple timing device such as used in the kitchen is set to ring after one hour. If the doctor himself cannot be present to read it, the blood level after one hour may be exactly marked by sticking a piece of adhesive paper to the tube, a task which is well within the competence of wife, secretary or even intelligent daily help, after instruction.

The upper limit of normal is 15 mm. in 1 hour. The other extreme is represented by myelomatosis where rates of over 100 mm. in *one half hour* are common.

*Suspected Infection, Acute and Chronic*

In any patient with a fever of unknown origin the appropriate investigations depend on the differential diagnosis; this of course varies from case to case. Pathological material, sent for bacteriological examination, should include urine in every case, with swabs, faeces, vomitus, sputum, pus or serous effusions where they were available. The initial routine investigation may also include (1) unclotted blood for full blood count, B.S.R. and biochemical tests, (2) clotted blood for serological tests and tests such as the flocculation reactions which are used in hepatitis, and (3) blood culture. The B.S.R. and the leucocyte picture may then suggest a group of infections which may be further investigated by serological tests such as the Paul Bunnell test for glandular fever, cold agglutination or M.G. streptococcus test in primary atypical virus pneumonitis, or the Brucella agglutination tests. In the case of suspected virus and rickettsial infections a specimen of serum secured early in the disease is of great value as it can be compared with a second specimen taken in convalescence. In virus serology such paired specimens are necessary in view of the great differences between individuals in the normal antibody titres. When blood culture is called for there is no substitute for an autoclaved syringe and needle in a sealed container which can be obtained from the laboratory before use. In carrying out the routine described 20 ml. of blood are desirable of which 10 ml. are added to a bottle of broth for culture, 5 ml. mixed with anticoagulant and 5 ml. collected in a plain bottle for serum. The blood in the needle is then used for making a blood film. If malaria is a possible diagnosis a thick smear is also made on the central one-third of a further side and *well dried*.

Urine from female patients must be taken by catheter if the bacteriological examination is important. In a male a mid-stream specimen passed directly into a sterilized bottle is satisfactory.

*Renal Diseases*

The finding of albuminuria should always call for a complete examination of the urine with microscopic and cultural examination of the deposit. If evidence of renal disease is present a blood urea estimation should be made. Further help may be given if the blood urea is combined with the urea clearance test. For this test two samples of urine are required each representing 60 minutes' renal output. The urine and blood samples are sent to the laboratory and the clearance as a percentage of normal can be calculated. It is essential that the bladder is empty before collection of the timed specimens is commenced and if the time intervals differ from 60 minutes the exact time in minutes should be stated no each bottle. The whole of the timed specimen should be sent because the calculation requires the renal output in ml. per minute. Other tests which may be helpful in chronic disease are serum cholesterol estimation and alkali reserve for which a clotted blood sample collected under a one-half-inch layer of liquid paraffin is required.

In prostatic cases, besides a urine examination valuable information may be given by B.S.R., haemoglobin, blood urea and acid and alkaline phosphatase determinations. They suggest whether or not there is a carcinoma with or without bone secondaries. Five ml. of E.D.T.A. (or potassium oxalate) blood are sufficient for all these tests. By having these tests carried out before the patient sees the surgeon, valuable time may be saved in arriving at a diagnosis and determining suitability for surgery.

*Investigation of Glycosuria*

The finding of a reducing substance in the urine calls for a blood-sugar estimation. A full glucose tolerance test is not always necessary in the first place. A single "sighting shot" may be all that is required and blood taken two hours after a good carbohydrate containing meal, e.g., breakfast will usually show whether the glycosuria is important. A urine specimen passed at the same time, examined for sugar and acetone, supplements the information given by blood sugar. One has seen a patient sent to the laboratory for glucose tolerance test showing clinical evidence of severe diabetes, in whom the fasting blood sugar was over 600 mg. per cent. In such a patient a dose of 50 g. of glucose would be ill-advised. If the result of the single test is in any way equivocal the full 2-hour glucose tolerance test should be carried out at the laboratory.

Glycosuria in pregnancy is usually physiological when it is due to milk sugar but if any doubt exists a single blood-sugar test as described is far more useful than a urine specimen for osazone or fermentation tests which are tedious to perform and often inconclusive where only a trace of reducing substance is present.

*Hepatic Disease*

When jaundice is the presenting feature a simple series of tests may be carried out to determine whether it is due to biliary obstruction or to infective hepatitis. Five ml. of clotted blood are required and a urine specimen should also be sent. The estimations used are serum bilirubin and serum alkaline phosphatase with serum colloidal gold, thymol turbidity and thymol flocculation tests.

The presence of bile in the urine will show that the icterus is of hepatic or obstructive origin and not due to haemolysis. In obstructive jaundice the alkaline phosphatase is usually much above normal (10 K.A. units) and the colloidal tests are usually negative for the first few weeks. In hepatitis the colloidal tests are strongly positive. The alkaline phosphatase is normal or moderately raised. In chronic cases where cirrhosis is suspected the plasma proteins should also be estimated.

### EMERGENCY SERVICE

Every hospital group now has a 24-hour emergency laboratory service. Arrangements differ in different areas but in general a practitioner who wishes to have an urgent specimen examined should phone the main hospital to which the area blood bank is attached. The technician or pathologist on call will then be informed by the hospital telephonist. In past years diphtheria swabs were the main business of after-hours work in laboratories but, with the virtual disappearance of that disease, first place has been taken by blood-transfusion requests. In most cases the patient should be transported to hospital but there are occasions when haemorrhage or shock is so severe that removal is impossible. A labelled specimen of the patient's blood (5 ml. clotted) is sent to the laboratory with a completed blood transfusion request form giving full name, age, sex, history of previous transfusions and of any transfusion reactions with, in the case of women the number of pregnancies, abortions and miscarriages. This form is a legal document and the person signing it is authorizing the transfusion and taking a share of responsibility for its consequences. If the case is one of dire emergency with the patient's life at stake, the messenger may take back a bottle of checked Group O Rh. negative blood to be given, unmatched, to the patient. Under no other circumstances can the risk of giving unmatched blood be taken. Meantime at the laboratory the patient's blood is grouped and further bottles are cross-matched by an emergency rapid procedure which takes approximately one hour from receipt of the specimen. Plasma should be given in the interim. In some centres no blood is issued without a full 2-hour cross match. If no flying squad service is available the practitioner would be well advised to call out a surgeon or obstetrician to supervise the management of the case.

In cases of coma where it is not possible to get the patient to hospital, blood may be sent for urea and sugar estimation, or the urine for barbiturates. Cerebrospinal fluids from cases of suspected meningitis are also a justification for emergency calls because of the importance of early specific treatment.

It should be realized, however, that specimens sent during laboratory hours will always receive fuller and more expert examination than those received at night. The laboratory works as a team and one man in the small hours of the morning cannot be expected to be as effective as a co-ordinated group of haematologists, biochemists and bacteriologists.

### CONCLUSION

The aim of this paper is not to teach clinical pathology to the practitioner who already uses the pathological services fully, but to encourage the non-user to "have a go". When next a difficult problem arises in his practice he should send an appropriate specimen (or the patient) to the laboratory and state his problem. It will be surprising if some help is not forthcoming; thus encouraged, he may take the laboratory into partnership in his practice, to the benefit of himself and his patient.

